# A Pathway to Psychological Difficulty: Perceived Chronic Social Adversity and Its Symptomatic Reactions

**DOI:** 10.3389/fpsyg.2018.00615

**Published:** 2018-04-27

**Authors:** Cody Ding, Jingqiu Zhang, Dong Yang

**Affiliations:** ^1^School of Psychology, Southwest University, Chongqing, China; ^2^Department of Educational Psychology, Research, and Evaluation, University of Missouri-St. Louis, St. Louis MO, United States; ^3^Key Laboratory of Cognition and Personality, Ministry of Education, Southwest University, Chongqing, China

**Keywords:** perceived chronic social adversity, chronic stress responses, emotion processing and regulation, individualized negative essentialism, resilience, cognitive control

## Abstract

In this paper, we attempt to predict and explain psychological maladjustment or difficulty. Specifically, we discuss the concept of perceived chronic social adversity, and we expect that such perceived chronic social adversity may potentially lead to chronic stress responses. Accordingly, we propose the symptomatic reactions of perceived chronic social adversity. We put forward a set of hypotheses regarding the relationships between perceived chronic social adversity and those chronic stress responses, and we further hypothesize a mediating role of individualized negative essentialism brought by perceived chronical social adversity. Resilience and individual differences in the ability to cope with perceived adversity are discussed. Future research and prevention need to pay more attention to effects of subjective personal experiences on psychological difficulty, focusing on the importance of exploring daily social experiences in improving cognitive construction processes and developing appropriate preventions.

## Introduction

The 21st century might be characterized by a faster-paced life, greater competitive pressure, and increased anxiety. Undoubtedly, most people, no matter how old they are, have encountered adversity or misfortune in their daily life, either perceived or real. At a psychological or behavioral level, the more important an issue is perceived to be, the more stress we feel. According to the perspectives of evolutionary psychology (Campbell et al., 1986; Kanazawa and Savage, 2009) or existentialism (Yalom, 1980), maintaining positive interpersonal relationships and personal excellence are the two most important issues for individuals. Thus, being frustrated by these two issues can put excessive pressure on individuals, and chronic exposure to negative daily encounters (such as deception, unfair treatment, being ignored, discrimination, bullying, or unemployment) might lead to psychological difficulties. Zhang et al. (2017) term this phenomenon as perceived chronic social adversity. It is therefore critical from both theoretical and practical view point to understand how psychological or behavioral problems could potentially develop following these daily social adversities. In this paper, we particularly focus on the concept of perceived chronic social adversity and its potential symptomatic sequelae. We also introduce a critical cognitive variable, *individualized negative essentialism*, that possibly mediates the relationship between perceived chronic social adversity (Zhang et al., 2017) and its symptomatic consequences.

## Perceived Chronic Social Adversity: Definition and Types

Everyone encounters challenges in their daily life and must cope with them using limited survival resources. Individuals need to integrate themselves into certain social networks to meet the needs of survival. This assertion can be supported by theoretical articulations. For example, the theory of social capital assumes people have access to resources inherent in their relationships (Bourdieu, 1980; Coleman, 1988). Their social resources, along with their personal resources, are significant in helping them achieve certain goals (Campbell et al., 1986). Similarly, from an evolutionary psychological perspective, social capital helps us, both directly and indirectly, to attain reproductive success (Kanazawa and Savage, 2009). According to the conservation of resource theory, people experience stress when circumstances threaten or result in loss of their personal and social resources (Hobfoll et al., 1990).

Based on the point above, we stipulate that being weak in social competition may be a result of an individual’s inability to obtain necessary and sufficient personal resources including respect, dignity, or social status. In this situation, individuals may become more eager to establish social networks and obtain social support. However, other than providing support, social networks may be characterized by negative events such as social undermining or negative social influence characterized by mistrust, hassles, criticism, and domination (Heaney and Israel, 2008), possibly leading to a loss of sense of security and an increase of anxiety about survival (Yalom, 1980).

According to Zhang et al. (2017), perceived chronic social adversity is a series of events that people perceived as stressful or overwhelming “because these events may occur repeatedly, continuously, or accumulatively during social interactions and social competition.” Whether or not a chronically occurring adversity event is assessed as very stressful or overwhelming by one person but not another depends on individuals’ previous personal experiences.

Although there may possibly be many adversity events, Zhang et al. (2017) suggest that perceived chronic social adversity mainly includes three types of social negative events. The first type is social exclusion or alienation. Macdonald and Leary (2005) defined social exclusion as the experience of being excluded from desired relationships or being devalued by partners, colleagues, or groups. Here, we focus on perceived deliberate social exclusion including the following aspects (either perceived or real): overlook, rejection, exclusion, unfairness, discrimination, disregard, suspicion, deception, or devaluation. These components are included as part of perceived social exclusion because we believe they share a common underlying theme. For example, unfair treatment may be perceived as not being valued or respected by others and thus may generally be perceived as a threat to social inclusion (Lind and Tyler, 1988); devaluation, being ignored, discrimination, disregard, suspicion, or deception may suggest lack of respect leading to exclusion. We think that the results of these aspects of exclusion might not be so dramatic, but if prolonged, exclusion can result in a variety of long-term negative effects on thought, emotion, and adjustment (Baumeister and Leary, 1995; Macdonald and Leary, 2005). Therefore, social exclusion is a vital part of perceived chronic social adversity.

The second type is being overly controlled in a close family or working relationship. One example of overcontrol can be seen in relationships with adults such as spouses, peers, friends, colleagues, or supervisors, involving emotional blackmail. As Forward and Frazier (1998, pp x–xii) stated, “emotional blackmail is a powerful form of manipulation in which people close to us threaten, either directly or indirectly, to punish us for not doing what they want.” People subjected to overcontrol may struggle to obtain love, respect, or trust and may have fewer opportunities to experience life as an individual. In addition, the inability to control their own destiny prevents them from using their interpersonal resources effectively and from obtaining social capital.

The third type is “weakness in social competition.” This component of adversity events involves disadvantage, powerlessness, or lack of voices in social interaction or competition for social resources. According to Festinger (1954), social competition is a basic drive that generally causes people to strive to do better and be more capable relative to comparative targets. Being equitable in social comparison may help people to protect and maintain their relative advantage in various social contexts such as organizational settings (Garcia et al., 2013).

As mentioned, perceived chronic social adversity is less intense and poses no direct, imminent threat to life or safety. However, because of its prominence in daily life and the substantial cumulative effect, the examination of perceived chronic social adversity and its symptomatic sequelae is theoretically and practically significant, and we believe it is a critical source of common psychological distress or difficulty.

## Symptomatic Sequelae of Perceived Chronic Social Adversity

In the following sections, we discuss potential symptomatic sequelae in the context of perceived chronic social adversity. This discussion is anchored around our literature review regarding the stress response system and sequelae of traumatic or stressful events.

Throughout history, humans have developed a highly effective protective system, which evolved in our original evolutionary environment. When perceiving a threat, people usually settle into a “hyperarousal” state in which they become hypervigilant to what is happening in their surroundings. As dominant emotions in response to threat, fear automatically prepares an individual to flee and anger automatically prepares an individual to fight. When human beings are attacked or traumatized, the sympathetic nervous system predominates, and the organism becomes extremely aroused and experiences general fear symptoms. In situations involving acute stress, particularly those that are life-threatening or extremely frightening, individuals may feel defenseless against danger and are more likely to become stuck in tonic immobility (i.e., fright) until the immediate danger has passed (Bloom and Farragher, 2010). However, when individuals are exposed to chronic stress, such as perceived chronic social adversity, the situation is different. In today’s world, we are confronted with psychological threats that may not be immediately resolved and may cause us to be constantly alert. Although these threats are not initially as astonishing or frightening as acute stressors, individuals may over-tax their perceptual and executive functions to prepare for fight or flight, particularly when these perceived threats are prolonged, leading to physical and mental depletion (McEwen, 2001, 2004; Charles et al., 2013) and feelings of being overwhelmed.

Based on these stipulations, we proposed a set of hypotheses regarding the relationships between perceived chronic social adversity and psychological difficulty:

(1) We hypothesize that people exposed to perceived chronic social adversity could experience affective domain problems, evidenced by heightened negative affect, high level of sensitivity, or emotional numbness.
(2a) We hypothesize that maintaining hypervigilance may be an emotion regulation strategy as a coping method for potential threats when individuals are consumed by worries or fears.
(2b) We hypothesize that perceived chronic social adversity may lead to anxious or depressive affects.
(2c) We hypothesize that the experience of perceived chronic social adversity has accumulative negative effects on individuals’ attention, interpretation, and memory.
(3) We hypothesize that perceived chronic social adversity may be directly or indirectly related to self-harm.
(4) We hypothesize that individuals with experiences of perceived chronic social adversity may suppress their anger or inhibit aggressive responses in a short term, but unresolved anger or frustration could accumulate and eventually explode, causing harm to others or self.

In the following sections, we discuss these hypotheses in more detail with respect to affect, perception, self-disturbance, and interpersonal functioning in the context of perceived chronic social adversity.

### Affective Domain Problems

Similar to Polani (2004) and Schauer and Elbert (2010) suggested that anxiety and panic may mimic the flight response in the face of ongoing threats and that these hyperactive syndromes entail depression. In other words, anxiety and depression may indicate somewhat different forms of responses to persistent and unresolved stressors (also see Tafet and Smolovich, 2004). Furthermore, chronic hyperarousal could finally deplete emotional resources and lead to emotional numbness (Litz et al., 1997).

Accordingly, we speculate that people exposed to perceived chronic social adversity could have differences in emotional processing and regulation according to their appraisals of their past experiences. We hypothesize that people exposed to perceived chronic social adversity could experience affective domain problems, evidenced by heightened negative affect, high level of sensitivity, or emotional numbness. This hypothesis has received some support from existing literature. For example, various types of social exclusion increase self-reported negative affect such as anxiety, depression, anger, shame (e.g., Leary, 1990; Chow et al., 2008; Meier et al., 2009; Yasin and Dzulkifli, 2009; Morrow et al., 2014), or emotional numbness (e.g., DeWall and Baumeister, 2006; Bass et al., 2014; Khaleque, 2014).

### Perceptual Overload

Social bonds can be highly effective when sharing information, food, or other resources needed to facilitate survival (Baumeister and Leary, 1995). Competitive advantage helps individuals obtain more resources (Buss, 1999). However, individuals who experienced perceived chronic social adversity may feel threatened in this regard. Findings from neuroscience studies (e.g., Meng et al., 2009) indicate that individuals are better attuned to negative stimuli. Thus, we hypothesize that maintaining hypervigilance may be an emotion regulation strategy as a coping method for potential threats when individuals are consumed by worries or fears. As a result, individuals may reduce the alarm threshold or detect threats as quickly as possible. Although these cognitive biases protected an individual from potentially fatal experiences in early human history (Baumeister et al., 2001), maintaining this same cognitive processing in today’s environment may be maladaptive.

Another perceptual overload is interpretation bias, which refers to the tendency to misinterpret ambiguous stimuli as negative or threatening. Previous experiences of rejection can predict the activation of defensive (anxious or angry) responses in ambiguous social situations (London et al., 2007). We argue that relationships characterized by inconsistent expectations, poor communication, or skirmish relationships can increase individuals’ appraisals of threat in ambiguous situations. We hypothesize that perceived chronic social adversity may lead to anxious or depressive affects. For instance, experimentally induced anxious or depressive moods are shown to result in higher levels of negative interpretation of ambiguous information relative to that observed among control subjects (Wright and Bower, 1992; Salmeto et al., 2011).

Third, threatening stimuli have been found to be effective in capturing attention, particularly when emotionally provocative (Öhman et al., 2001). According to Schauer and Elbert (2010), individuals remain hypersensitive to threat only when negative information is highly self-relevant and presented for a long period of time (Mogg and Bradley, 2005). We hypothesize that experiences of perceived chronic social adversity have accumulative negative effects on individuals’ attention, interpretation, and memory.

### Self-Disturbance Domain

People often struggle to adapt to cultural norms and reconcile their own desires and feelings with the values and expectations of others, such as peers, schools, companies, or the broader society. Through various interpersonal relationships and multiple social comparisons, people learn who they are and gradually develop positive or negative views of themselves. Positive self-expansion (e.g., increasing one’s status, economic position, or affiliation; Beck and Haigh, 2014) and perception of competence and acceptance from others (Lachowicz-Tabaczek and śniecińska, 2011) are suggested to be associated with increased self-esteem. In contrast, perceived chronic social adversity could lead to negative beliefs about the self. For example, social exclusion and abusive supervision have been found to decrease self-esteem or poor self-concept (Leary et al., 1995; Burton and Hoobler, 2006). Furthermore, perception of the comparison target as better than oneself also decreases self-esteem (Locke, 2003).

Low self-esteem or self-blame for personal failure or setbacks have been found to be effective in predicting current and future self-harm, suicide ideation, and most suicide attempts (Baumeister, 1990; Bhar et al., 2008; Phillips et al., 2013). In addition, a direct relationship has been observed between stressful events and self-harming related thoughts and behaviors. For example, exposure to social exclusion was significantly associated with self-harm (Wu et al., 2013) and increased death-related thoughts (Steele et al., 2015). Similarly, workplace bullying has predicted subsequent suicidal ideation (Nielsen et al., 2015). Accumulated exposure to various types of interpersonal adversity has been associated with dysfunctional avoidance indicated by suicidality, substance abuse, dissociation, and problematic behaviors such as self-injury and dysfunctional sexual behavior (Briere et al., 2010). We hypothesize that perceived chronic social adversity may be directly or indirectly related to self-harm, which also constitutes an emotion regulation strategy (e.g., Chapman et al., 2006).

### Poor Interpersonal Functioning

Satisfactory relationships may require reciprocal social interaction in an equitable or balanced manner. Perceived chronic social adversity may disturb this social homeostasis. Once negative beliefs regarding the self (e.g., clumsy, unpopular, or incapable) or others (e.g., untrustworthy, dangerous, insincere, or inaccessible) are formed and developed, individuals decide whether and how to engage in social interaction. Considering social exclusion, when the perceived possibility of relational alternatives is high, individuals might merely withdraw from people or groups that excluded them. But continuous or repetitive exclusion may deplete individuals’ psychological resources (Williams, 2007; Richman and Leary, 2009), and they could perceive their value to others as low and their presence as a burden (Allen and Badcock, 2003).

Numerous studies have shown that social exclusion lead to antisocial behavior (e.g., Twenge et al., 2001; Leary et al., 2003) or decreased prosocial behavior (e.g., Twenge et al., 2007; Poon et al., 2013). When individuals’ needs for respect, fairness, autonomy, or recognition are thwarted (Warburton et al., 2006; Williams, 2007; Richman and Leary, 2009), they may be prone to think, feel, or behave in an antisocial manner. For example, based on a case analysis of 15 school shootings in the United States, Leary et al. (2003) suggested that 87% of school shootings are characterized by chronic social exclusion combined with a specific, usually recent, rejection experience. A more recent mass shooting that occurred at Marjory Stoneman Douglas High School in Parkland, Florida on February 14, 2018 may also speak to this point. While we deeply feel pain about the loss of lives, we also know that the 19-year-old gunman has experienced significant trauma, whether it is real or perceived. Similar situations could happen in overcontrolled relationships where individuals who perceive themselves to be at a competitive disadvantage or experience competition failure are more likely to show increased aggression and engage in antisocial behavior (Harris and Fisher, 1973; Sagar et al., 2011). Although some evidence has revealed that higher social status (Diekmann et al., 1996) and better performance are positively related to aggression (Muller et al., 2012), these studies did not consider aggression toward others who were not involved in the social comparison. We hypothesize that individuals with experience of perceived chronic social adversity may suppress their anger or inhibit aggressive responses in a short term, but unresolved anger or frustration may accumulate and eventually explode, causing harm to others or self.

## Individualized Negative Essentialism: A Crucial Mediator Between Perceived Chronic Social Adversity and its Symptomatic Sequelae

In this section, we hypothesize that the potential effect of perceived chronic social adversity on psychological difficulty is at least partially mediated by individualized negative essentialism. We discuss this hypothesis in more detail below.

Early in development, individuals begin to use categories to learn about the world. By sorting out the diverse entities into separate classes or categories based on their personal experiences, individuals can obtain a manageable system and quickly respond to different types of objects or situations, which is beneficial to their survival (e.g., Ashby and Maddox, 2005). However, when individuals excessively relying on these categories of experiences, or regard the boundaries around the categories as closed and fixed, they may obscure and overlook the complexity of what may actually be happening (Jones, 2009).

For example, feeling being ignored or disrespected in a particular group, whether it is true or perceived, individuals may generalize this information to the entire category (e.g., people of this kind are not nice in general) or make a classification of self (e.g., I am just the sort of person who can be ignored) and use this information to guide future behavior (e.g., become bitter or avoid similar situations).

A critical assumption that underlies this form of reasoning is the belief that members of a category share the same or similar underlying and consistent nature (we term this subjective belief as “individualized essence”) and so they are highly similar to one another. Therefore, if individuals are constantly exposed to perceived chronic social adversity, they may tend to generalize those experiences to a broad context and thus judge the self, others, or situations in a fixed and negative way. This is what we called *individualized negative essentialism*. Through the process of individualized negative essentialism, people who experience perceived chronic social adversity could develop ill-fitting means of processing information and a biased view of the world (including self, others, or events) which is usually accompanied by undesirable psychological and behavioral responses.

According to cognitive theorists, social information processing, particularly in construal processes, plays an important role in the development and maintenance of various forms of psychopathology (Treat et al., 2002). According to the generic cognitive model of Beck and Haigh (2014), information processing relies on two interactive subsystems: the automatic processing and reflective processing systems. The automatic processing system is initially triggered by events that signal personal threat, gain, or loss. It fits incoming data into gross categories and is likely to produce errors. The reflective system, which is more objective and refined, usually reforms or corrects the subjective meanings produced by the automatic system. However, when negatively biased automatic processing is not corrected by reflective processing, cognitive vulnerability to psychological distress occurs (Beevers, 2005).

As a typical exemplar of cognitive theory, Kelly’s (1955) personal construct theory has more in common with contemporary cognitive science relative to numerous other cognitive theories in psychology (Treat et al., 2002). This theory suggests that the meanings of events are subject to various types of construction and are therefore open to reconstruction when predictions are disconfirmed (Kelly, 1955). Individuals develop a system of constructs based on their unique experiences which is then used to categorize their experiences and interpret, predict, and respond to future experiences (Butt and Parton, 2005; Moes-Williams, 1997, unpublished). Thus, we stipulate that individuals who repeatedly experience perceived negative events may polarize their experiences in extreme ways, leading to difficulties in emotion processing and regulation. Based on these theoretical assumptions, we hypothesize that the potential effect of perceived chronic social adversity on psychological difficulty is at least partially mediated by individualized negative essentialism.

## Conclusion and Future Directions

This paper discusses the fundamental ideas of perceived chronic social adversity and its symptomatic consequences. Our goal is to explain a pathway to mental suffering such as anxiety, depression, self-harm, or antisocial behaviors. As Beck and Haigh (2014) suggested, environmental events influence each stage of life development and determine the nature of the schema content and psychological maladjustment. While more attention is paid to traumatic events, studies usually focus less on daily social adversity, especially how people construe their daily social adversity. Although previous studies have indicated the strong association between chronic environmental stressors and mental health (e.g., Evans et al., 2013; Arnold et al., 2014; Orcutt et al., 2014), this paper particularly focuses on highlighting the importance of personal experiences as a source of potentially perceived trauma and needs for prevention before occurrence of catastrophic events.

We conceptualize perceived chronic social adversity as social events that may be perceived as stressful or overwhelming. Though daily social adversity appears to be less intense than traumatic events, it may potentially cause excessive stress because of its chronicity, harming our psychological well-being. Muraven and Baumeister (2000) suggest attempts to cope with stress and regulate negative affect requires self-control, which may consume a limited resource. Accordingly, we expect continuous effort of self-control may decrease over time if individuals are feeling stuck in perceived chronic social stress. In other words, individuals may be subjectively overwhelmed by social adversity if it is prolonged enough. In such cases, greater distress can be expected to be brought on by these actually more controllable things (Caston and Frazier, 2013).

The literature review offers unsystematic but extensive empirical support for symptomatic sequelae among people exposed to perceived chronic social adversity. These chronic stress responses are mainly reflected by ill-fitting emotion processing and regulation, such as emotional dysregulation of negative affect, perception overload, and maladaptive coping strategies. We put forward a set of hypotheses regarding the relationships between perceived chronic social adversity and psychological difficulty and hypothesize that those chronic stress responses can be mediated by individualized negative essentialism, which is largely triggered by cumulative or prolonged negative events. As an ill-fitting emotion processing and regulation mechanism, individualized negative essentialism can help us better understand a developmental pathway between perceived chronic social adversity and maladjustment.

We also need to point out that not everyone who experiences perceived chronic social adversity develop individualized negative essentialism and psychological difficulty. Issues of resilience need to be recognized; that is, why can most people cope well? What are the mechanisms or factors that allow individuals to manage successfully or bounce back from perceived chronic social adversity? For example, the ability to keep a sense of hope that provides meaning to adversity may help individuals to get up each day and harness resources applicable toward getting a better future (Panter-Brick, 2014). There are a lot of different factors that might make individuals more resilient (for a review, see Bonanno et al., 2011) and we need to examine factors that can best mediate or moderate the relations between perceived chronic social adversity and mental illness. Bonanno and Burton (2013) suggested three key elements to resilience: (1) how the situation is perceived (situation sensitivity), (2) a set of behaviors, and (3) the ability to correctly re-group information. We believe that there are strong associations among these three elements, our conceptualization of individualized negative essentialism, perceived chronic social adversity, and mental distress.

Early-stage prevention of psychological difficulty is extremely critical, and our theoretical stipulations in this paper may provide a useful theoretical and therapeutic framework for understanding the key factors contributing to psychological difficulty. Of course, empirical studies are really needed to provide evidence for these relationships among perceived chronic social adversity, individualized negative essentialism, and psychological difficulty. A key point is we should address daily social adversity history of individuals and commit to appreciating their constructions of events in order to circumvent potentially serious consequences.

## Author Contributions

CD and JZ: concept and write-up of the manuscript. CD, JZ, and DY: critical revision/review.

## Conflict of Interest Statement

The authors declare that the research was conducted in the absence of any commercial or financial relationships that could be construed as a potential conflict of interest.
